# Influence of Plant-Based Biostimulant (BORTAN) on Qualitative and Aromatic Traits of Rocket Salad (*Diplotaxis tenuifolia* L.)

**DOI:** 10.3390/plants12040730

**Published:** 2023-02-07

**Authors:** Livia Malorni, Rosaria Cozzolino, Anna Magri, Luigi Zampella, Milena Petriccione

**Affiliations:** 1 Institute of Food Science, National Research Council (CNR), Via Roma 64, 83100 Avellino, Italy; 2 Department of Environmental, Biological and Pharmaceutical Sciences and Technologies (DiSTABiF), University of Campania Luigi Vanvitelli, Via Vivaldi 43, 81100 Caserta, Italy; 3 Diachem S.p.A., Via Mozzanica 9/11, 24043 Caravaggio, Italy; 4 Council for Agricultural Research and Economics (CREA), Research Center for Olive, Fruits, and Citrus Crops, Via Torrino 3, 81100 Caserta, Italy

**Keywords:** *Diplotaxis tenuifolia* L., plant-based biostimulant, sustainable horticulture, nutritional quality, volatile organic compounds (VOCs)

## Abstract

In this study, the influence of a new plant-based biostimulant (Bortan) on physiological and aromatic traits of rocket (*Diplotaxis tenuifolia* L. var. Pamela) was monitored by evaluating physico-chemical parameters (fresh and dry weight, leaf color and chlorophyll content) and biochemical traits (total phenolic compound (TP), total flavonoids (TF), ascorbic acid (AA) and antioxidant activity (AOX). Volatile profiles were also analyzed by headspace solid-phase microextraction coupled to gas chromatography–mass spectrometry, allowing the detection of 32 volatiles belonging to 5 chemical classes. Compared to the control, Bortan application enhanced leaf pigment content, including chlorophyll a, b and carotenoids (+10%, +16% and +28%, respectively) and increased TP (+34%), TF (+26%), AA (+19%) amonts and AOX value (+16%). Principal component analysis revealed a significant discrimination between the two samples. Specifically, treated samples were mainly associated with ”green-leaf” volatiles, namely hexanal and 2-hexenal, 3-hexenal and 1-penten-3-one, while control rocket was directly correlated with several alcohols and to all isothiocyanates, associated with the sulfur-like odor of rocket. These findings can add further support, both for farmers and the agro-food industry, in choosing PBs as a new and sustainable practice in complementing enhanced yields with premium-quality produce. To confirm these preliminary data, further experiments are needed by enlarging the sample size, testing different concentrations of Bortan and/or using other food crops.

## 1. Introduction

Earth’s population has doubled since 1960 with a consequent higher-than-expected increase in food demand and production [1]. To face the growing global demand for food, agriculture has unsustainably responded by consuming large amounts of natural resources: 70% of global fresh water (FAO, 2017) and approximately 40% of arable land [2]. Moreover, about 14% of the world’s greenhouse gases (GHG), including methane, nitrous oxide and carbon dioxide, are released by agricultural practices [2], triggering a boomerang effect of being one of the main contributors to climate change and, simultaneously, being influenced by it [2]. 

Consequently, in the last decades, the development of eco-friendly methodologies to enhance the sustainability of agricultural systems, avoiding the generation of further environmental pollution, has been one of the crucial challenges for agricultural research. In this context, there has been an increasing interest in natural plant biostimulants (PBs), a new promising and environmentally friendly generation of products which have been documented to extend beneficial effects on crop productivity and quality, also improving plant tolerance against a wide range of abiotic stresses [3]. Over the past twenty years, research and applications of PBs in agriculture have increased to limit the number of conventional pesticides and fertilizers introduced into the environment, reducing the pollution of soil, water and air [4]. Vegetal-based biostimulants can either directly influence the metabolism and the physiology of plants or indirectly influence these attributes by increasing soil conditions through regulating the microflora, which can positively affect plant development [5].

Regulation (EU) 2019/1009 [3] has defined plant biostimulants as follows: “A plant biostimulant shall be an EU fertilising product the function of which is to stimulate plant nutrition processes independently of the product’s nutrient content with the sole aim of improving one or more of the following characteristics of the plant or the plant rhizosphere: (i) nutrient use efficiency, (ii) tolerance to abiotic stress, (iii) quality traits, or (iv) availability of confined nutrients in the soil or rhizosphere” (EU, 2019).

PB formulations, defined according to their agricultural functions claims, contain a mix of bioactive molecules of natural origin working in synergy, including humic and fulvic acids, protein hydrolysates of animal and vegetal origin, extracts of macroalgae seaweeds and silicon, along with beneficial microorganisms, such as arbuscular mycorrhizal fungi (AMF) and N-fixing bacteria of strains of the genera *Rhizobium*, *Azotobacter* and *Azospirillum* [3]. Several studies have reported the possibility of developing efficient PBs from agro-food and industrial waste [3,6], demonstrating that PBs can open new opportunities in a full circular economy approach, thanks to the lack of pesticides and heavy metals, low-cost storage and sufficient accessibility throughout the entire year [3]. The European Union, through the “Green Deal” action, promotes all activities that lead to sustainability. In the context of plant nutrition, plant-based biostimulants could reduce the use of fertilizers and plant growth regulators, allowing for more sustainable agriculture [7,8].

PB management is important as their action strictly depends both on different species and/or cultivars of the same species, and on environmental condition, dose and application time [5]. PBs can be sprayed on either the soil or the leaves of plants, based on their composition and desired outcomes, improving plants’ resilience to various biotic and abiotic stresses [9]. They trigger biochemical and physiological processes (photosynthesis, nutrient and water uptake and assimilate partitioning) in host plant tissues and cells, modulating the synthesis and signaling pathways and inducing hormonal responses involved in plant growth and development, thus boosting crop quality and yield [4,6,10].

*Diplotaxis tenuifiolia* L., commonly known as wild rocket, roquette, arugula or rucola, belongs to the Brassicaceae family which includes many edible vegetables, such as *Brassica oleracea*, (broccoli, cabbage, cauliflower) *Raphanus sativus* (radish) and *Sinapis alba* (white mustard), that are well-known sources of health-promoting biochemical com-pounds [11]. Wild rocket is high in biologically active components, including ascorbic acid, carotenoids, fibers, glucosinolates and phenolic compounds. This crop is also very appreciated for its rich pungent taste and strong characteristic flavor [12]. Accumulating scientific evidence has demonstrated that odor and flavor perceptions, which greatly influence consumers’ preference, are the result of a synergistic combination of a high number of volatile organic compounds (VOCs) [13,14,15]. In this regard, the distinctive pungent flavor of fresh rocket is not due exclusively to glucosinolates and their break-down products (isothiocyanates, ITCs), as other VOCs were also showed to be particularly significant, including some C6 aldehydes and alcohols (hexanal, *trans*-2-hexenal, *cis*-3-hexen-1-ol and *trans*-2-hexen-1-ol) being responsible for green notes, and benzaldehyde and *trans, trans*-2,4-heptadienal being described as responsible for a nutty almond aroma [16]. 

Similar to other leafy vegetables, rocket is recognized as a nitrate hyper-accumulating crop. As nitrate accumulation relies on nitrogen availability and on particular environmental conditions, scientific attention has focused on a suitable handling of nitrogen fertilization, also by using alternative strategies, including the employment of natural PSs [5]. Several authors have described positive effects on yield and on growth parameters of a wide range of fruit and leafy vegetables, including rocket, following the application of PBs [5,17,18]. 

Considering the above remarks, in this study, the effects of a new formulated plant-based biostimulant, called Bortan, were investigated on several physico-chemical parameters, biochemical features and VOC profiles in wild rocket (*Diplotaxis tenuifolia* L.) in soil culture.

## 2. Results and Discussion

### 2.1. Colorimetric Parameters and Leaf Chlorophyll Content 

Color, which is correlated with leaf chlorophyll concentration, is a significant quality parameter with a direct effect on the visual appearance of leafy vegetables. Chlorophyll is an important pigment and a quality trait that gives some indication of the physiological status of the plant. Since most of the plant’s nitrogen is incorporated in chlorophyll, this molecule can be considered as an indicator of the nutrient status of the plant, in addition to being responsible for the visual appearance of leafy vegetables and consumer preference [19,20]. Green leafy vegetables can show color changes after harvest due to high biological variance and the product’s heterogeneity [21]. In this study, colorimetric parameters showed statistically significant differences in rocket under biostimulant application. In fact, compared to the untreated samples (a*: −7.25 ± 0.34; b*: 15.09 ± 0.54), the green intensity (a*: −8.29 ± 0.49; b*: 19.53 ± 1.36), with lower a * values and higher b * values, was observed to increase in treated rocket leaves (Table 1). On the other hand, previous studies on rocket leaves have demonstrated that the application of a biostimulant based on tropical plant extracts and Auxym and legume-derived protein hydrolysate (LPDH) showed no effect on leaf color [5].

Photosynthetic pigments are among the most significant factors that affect the photosynthesis process, primary plant production and, subsequently, the postharvest life of leafy vegetables [22]. 

**Figure 1:**
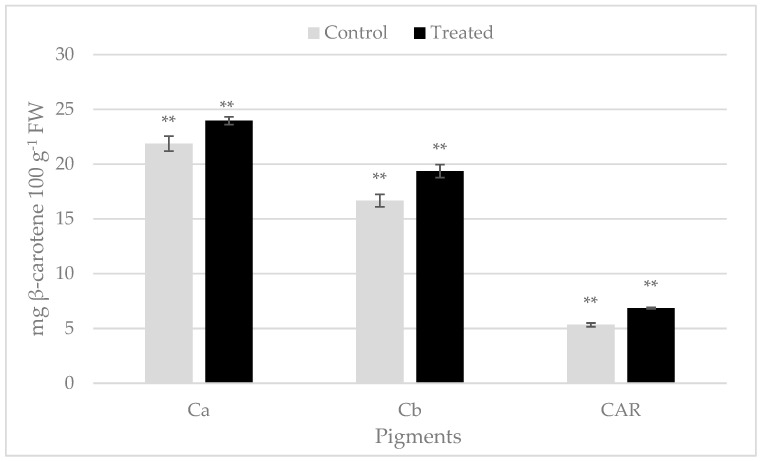
Photosynthetic pigments in the rocket leaves treated with Bortan (Treated) and in the untreated samples (Control) (Ca: chlorophyll a; Cb: chlorophyll b; CAR: total carotenoids). The grey bars indicate the control samples and black bars the treated ones. Pigment content is expressed as mg β-carotene 100 g^−1^ FW; ** significant for *p* ≤ 0.01.

Chlorophyll and carotenoid contents in rocket leaves exhibited statistically significant differences following biostimulant treatment. The content of chlorophyll a showed a value of 21.87 ± 0.68 mg 100 g^−1^ FW in untreated rocket and 23.97 ± 0.3 mg 100 g^−1^ FW in treated leaves (Figure 1). The amount of chlorophyll b was 16.66 ± 0.57 mg 100 g^−1^ FW in the control sample, while it was 19.36 ± 0.60 mg 100 g^−1^ FW in treated rocket (Figure 1). Total carotenoids also turn out to be higher in treated samples than in untreated ones (Figure 1).

Previous studies have demonstrated that the content of chlorophyll a and b and of carotenoids were positively influenced by plant biostimulant application (LDPH) in rocket, baby lettuce, sweet corn and eggplant [5,23,24]. This could be due to an increase in the amino acid content in LDPH-treated plants, which can enhance photosynthetic pigment production [5]. Furthermore, an increase in photosynthetic pigments has been reported in wild rocket treated with several PBs [25,26,27]. 

Similarly, carotenoids, accessory pigments in light harvesting, that protect the chlorophylls from photooxidation, displayed an increase in plants following PB applications [28,29].

### 2.2. Bioactive Compounds and Ascorbic Acid Content

Antioxidant molecules present in leafy vegetables are widely reported to extend health benefits [30]. In particular, phenolic and flavonoid compounds can have a central role in disease prevention. Phenolic compounds are secondary metabolites invariably found in plant-based food. These phytochemicals arise from a variety of biological pathways, including the pentose phosphate, shikimate, and phenylpropanoid pathways [31]. In this study, the biostimulant treatment influenced the total polyphenol content, as treated rocket showed the highest value of TP (91.94 ± 5.04 mg GAE 100 g^−1^ FW) with respect to the untreated samples (68.56 ± 3.45 mg GAE 100 g^−1^ FW) (Table 2).

Flavonoids are thought to be the main phenolic compounds in food [32]. Control samples contained 81.56 ± 2.29 mg CE 100 g^−1^ FW of TF, while treated rocket leaves showed a value of about 102.51 ± 7.00 mg CE 100 g^−1^ FW (Table 2). These findings are in line with previous studies which reported that different PBs determined an increase in TP content in several horticultural crops, including wild rocket [27], spinach [33,34], cucumber [35] and common beans [36]. 

Vegetables contain the highest amount of ascorbic acid (AA) when they are in the fresh activated state, while any handling tends to decrease the original content of this molecule [37]. In our study, AA production increased with biostimulant application. In fact, AA presented a higher value in the rocket samples treated with the biostimulant (16.63 ± 0.69 mg AA 100 g FW) compared to the untreated leaves (14.04 ± 0.05 mg AA 100 g FW). These results are in agreement with previous studies assessing that in wild rocket, AA content was enhanced by the application of PBs [5,18]. 

Rocket salad has been reported to possess significant levels of bioactive components and high antioxidant activity [38]. In the present study, AOX was higher in treated samples (9.99 ± 0.14 µmol TE g FW) compared to the untreated (8.61 ± 0.49 µmol TE g FW) ones, in line with Caruso et al. (2019), who determined that PB application increased antioxidant activity in wild rocket [18]. 

### 2.3. Volatile Compounds Analysis

Overall, 32 volatile compounds were identified or tentatively identified by the HS-SPME/GC–MS analysis of treated and untreated rocket leaves, including ketones (4), aldehydes (7), alcohols (8), glucosinolate hydrolysis products (GHPs) (8) and others (5). HS-SPME/GC–MS semi-quantitative data (RPA%) were treated with a two-sample *t*-test using MetaboAnalyst v5.0 to evaluate the effect of the application of the biostimulant on the detected volatiles. The obtained results showed significant differences in the volatile profiles, in both quantitative and qualitative terms, between the treated and control rocket, except for anisole (O4), as reported in Table 3, which also lists VOC abbreviation codes, experimental and literature Kovats index values and methods of identification. 

The VOC profile of the rocket samples was in a good agreement with previously reported data as, among the revealed volatiles, 26 compounds, including 3-pentanone (K1), 1-penten-3-one (K2), 3-octanone (K3), 6-methyl-5-hepten-2-one (K4), hexanal (Ald1), *cis*-3-hexenal (Ald2), 2-hexenal (Ald3), octanal (Ald4), nonanal (Ald5), 2,4-heptadienal (Ald6), 1-Penten-3-ol (Al1), *cis*-2-penten-1-ol (Al2), 1-hexanol (Al3), *trans*-3-hexen-1-ol (Al4), *cis*-3-hexen-1-ol (Al5), *trans*-2-hexen-1-ol (Al6), methyl TC (GHP1), 3-butenyl ITC (GHP2), pentyl ITC (GHP3), 4-methylpentyl ITC (GHP4), hexyl ITC (GHP5), 3-methylthiopropyl ITC (GHP6), 2-ethylphenyl ITC (GHP7), 2-ethylfuran (O1), *cis*-3-hexen-1-ol acetate (O2) and 5-methylhexanenitrile (O5), were already detected [11,16,39,40,41,42,43,44]. Table 3 indicates that three VOCs, namely K4, Ald4 and GHP2, were detected only in the control samples.

Alcohols were the most representative compounds in the control sample, accounting for about 48.6% of the total VOCs, with *cis*-3-hexen-1-ol (Al5) as the main constituent (28.8% of the total volatiles) (Table 3). On the other hand, in the treated rocket, aldehydes were the predominant volatile metabolites, with 2-hexenal (Ald3) as the most abundant compound (58.2% of the total volatiles) (Table 3). It is worth mentioning that very recently, Bell et al. (2021) observed that even when detected at high abundance, *cis*-3-hexen-1-ol can impart only feeble fresh green notes in rocket leaves, while *trans*-2-hexenal, if present in relatively less abundance, can produce a rather strong flavor, described as fresh, leafy, green and apple-like [44]. 

In both treated and untreated samples, isothiocyanates (ITCs), the hydrolysis products of glucosinolates by myrosinase activity following tissue crushing, are the third class of VOCs in terms of quantity (15,1 and 8,8% of the total volatiles, in the treated and untreated rocket, respectively) (Table 3). These metabolites, which are mainly responsible for the typically pungent and Brassicaceae-like odor of fresh rocket leaves, were described to extend potential benefits to human health [16,40].

### 2.4. Correlation Analysis among Physico-Chemical, Biochemical and VOCs Data 

**Figure 2:**
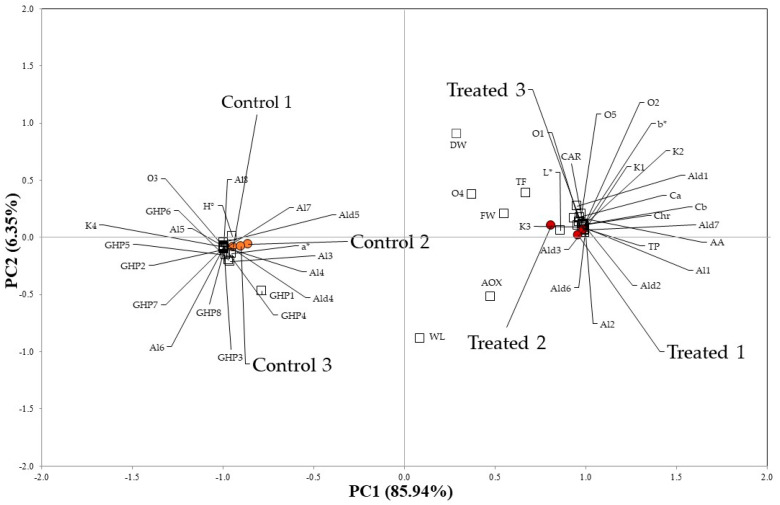
Principal component analysis performed on Ca, Cb, CAR, TP, TF, AA, AOX and on the semi-quantitative data (% RPA) of all VOCs detected in the rocket leaves treated with Bortan (Treated) and in the untreated samples (Control) (Codes are reported in the text and in Table 1, Table 2 and Table 3).

Exploratory principal component analysis (PCA) experiments were carried out on the dataset, composed of six observations (two biological samples as technical triplicates), three photosynthetic pigments (Ca, Cb, CAR), three biochemical parameters (TP, TF, AA), the AOX, and the HS-SPME/GC–MS semi-quantitative data of the 32 detected VOCs (Table 3), to investigate possible significant correlations in untreated and treated rocket leaves (Figure 2).

The two components explained 92.3% of the variation in the dataset, since PC1 and PC2 accounted for 85.9% and 6.4% of the total variance, respectively. In the PCA plot, treated and control samples were clearly separated: rocket leaves grown in presence of the plant-based biostimulant are located in the right part of the score plot, with both PC1 and PC2 being positive (Figure 2). In contrast, control rocket samples are located in the left part of the PCA score plot, with both PC1 and PC2 being negative (Figure 2). These samples showed a significant correlation with L*, b*, FW, Chr, TP, Ca, Cb, CAR, AA and with eighteen VOCs: two aldehydes (Ald4 and Ald5), six alcohols (Al3-Al8) one ketone (K4), all eight GHPs (GHP1-GHP8) and the ester cis-3-hexen-1-ol acetate (O3). C6 compounds (Al3-Al8, O3) are produced as plant defense metabolites following leaf injuries by the lipoxygenase (LOX) pathway from fatty acids. They are described as green-leaf-scented molecules [16]. 

Regarding the GHPs, the amounts of each of these metabolites are statistically higher in the control samples compared to the treated rocket (Table 3). 

Very recently, Bell et al. (2021) investigated the flavor profiles of four Brassicaceae species, including rocket salad, to determine the odor-active volatiles in the headspace of the studied crops [44]. In this study, new odor descriptions were possible to associate with several VOCs, including the different ITCs detected. Specifically, methyl TC (GHP1) was found to impart to rocket salad sulfury—but also oniony—notes with a medium intensity, while 3-butenyl ITC (GHP2), previously described as aromatic and pungent, was noted to also impart green notes at a medium–weak intensity [44]. It is noteworthy that among GHPs, 3-butenyl ITC (GHP2), detected only in the untreated leaves (Table 3), was reported to have cytotoxic potential against the human prostate cancer cell line. As it is able to induce cell death via apoptosis, it has been suggested to propose GHP2 as an anticancer compound [45]. 

Pentyl ITC (GHP3), already indicated as green, has also been recently associated with the cabbage-like and strong rotten odor in rocket salad, while 4-methylpentyl ICT (GHP4) has been characterized by an odor of weak intensity described as “musty” for the first time [44]. Moreover, benzyl ITC (GHP7), defined to have a rotten-grass, cooked smell of medium–weak intensity, has been indicated as an odor-active constituent even at very low concentration [44].

In a previous study, Bell et al. (2017) reported that hexyl-ITC (GHP5) was significantly correlated with the aroma perceptions of mustard and sulfur, suggesting that this compound heavily contributes to rocket odor traits, despite the low relative amount within the VOC profile [46]. Finally, 3-methylthiopropyl ITC (GHP6) was recognized among the odor-active compounds responsible for the radish and rocket-like notes in rocket, while 2-ethylphenyl ITC (GHP8), observed by Raffo et al. (2008) in the leaves of *Diplotaxis tenuifolia* L., cv Grazia, was not recognized as an odor-active compound [42].

6-Methyl-5-hepten-2-one (K4), only detected in the control sample, was previously found to impart a citrus odor in wild rocket (Table 3) [16]. Recently, in addition to this sensory descriptor, it has been also reported to have a medium–strong floral and perfume-like aroma [44]. Other minor volatiles positively correlated with the control sample included octanal (Ald4), a derivative of oleic acid oxidation reported to impart green notes in rocket [16,43,44]. Ald4 was only identified in the untreated rocket leaves (Table 3).

Lastly, it should be pointed out that Bell et al. (2021) have noted that several unknown compounds and components regularly observed in rocket flavor, including 1-penten-3-ol and hexanal, generated a pungent aroma of an intensity comparable to that of the GHPs, suggesting that these sulfur metabolites may not be the only constituents responsible for this attribute of rocket flavor [44].

Treated rocket leaves samples are directly associated with a* and H°, in line with a higher green intensity of the leaves, and with thirteen volatiles: three ketones (K1-K3), five aldehydes (Ald1-Ald3, Ald6 and Ald7), two alcohols (Al1 and Al2) and three others (O1, O2 and O5).

In rocket, aldehydes, alcohols and ketones are reported to show a high degree of association with some flavor, taste and mouth-feel features, including sweet, stalky and green [44]. Among the several aldehyde components significantly correlated with the treated samples, hexanal (Ald1) and 2-hexenal (Ald3) are associated with the green aroma perception of rocket salad [46]. At the same time, Ald3 is also reported to give herbal aroma impressions, while Ald1, previously described to contribute to the fatty side-notes [39], as mentioned above, has been recently reported to impart pungent notes of relatively high intensity in rocket [44].

C5 compounds (K1, K2, Al1, Al2), which are synthesized through the LOX pathway from linolenic acid, are always statistically higher in biostimulant-treated rocket leaves (Table 3) [44]. Specifically, among C5 compounds, in rocket salad, 3-pentanone (K1) was associated with ethereal notes, 1-penten-3-one (K2) was found to be related with pungent, ethereal and spicy odors, *cis*-2-penten-1-ol (Al2) was linked with ethereal and fruity notes and 1-penten-3-ol (Al1), described as having sweet scents, has very recently been shown to produce a pungent aroma [16,44,46]. It is important to highlight that in rocket, two ”green-leaf” volatiles, namely 3-hexenal (Ald2) and 1-penten-3-one (K2), have been reported as having an odor potency comparable to GHPs within the volatile bouquet [44]. 

Other minor volatiles positively correlated with the treated samples include the following secondary lipid oxidation products: 2,4-heptadienal (Ald6), with sweet, fruity citrus/melon odor with spice notes, decanal (Ald7), a sweet, aldehydic and citrus compound, and 2-ethylfuran, with an ethereal odor type [47].

Bell et al. (2017), studying seven rocket salad accessions, have noted that there is a possible association between the relative amounts of “green-leaf” volatiles and the pungency perception triggered by sulfur-containing metabolites, including ITCs. As both the volatile families have an active part in plant defense, this association could be translated in an evolutionary strategy to promote one biosynthetic pathway over the other and vice versa. The regulation of ITC formation and of the LOX pathway for “green-leaf” volatiles production may be in charge of the equilibrium between ITCs/sulfur volatiles and “green-leaf” volatiles synthesis. The relative contents of VOCs between these two pathways are probably the crucial factor in rocket sensory traits [46].

## 3. Materials and Methods

### 3.1. Plant Material and Growth Conditions

Rocket seeds (*D. tenuifolia* (L.) DC. “Pamela”) were purchased from Biogya (Viterbo, Italy). The seeds were sown in a polyethylene greenhouse (7.20 × 50 mt) in the commercial farm “Monetti”, Battipaglia, Salerno, Italy (40°33′ N; 14°55′ E; 78 m a.s.l.).

The treatments consisted of control plants, watered at 100% of field capacity, and of plants supplied with Bortan, provided by the company Diachem, Caravaggio (BG), Italy (https://www.diachemitalia.it/prodotti/biostimolanti-e-fertilizzanti-speciali/bortan accessed on 23 January 2023). Bortan is a new nitrogen organic fertilizer with 10% humic extracts rich in tannins obtained from natural-origin products in liquid form. Bortan has complexing properties that enhance growth of the root system and the assimilation of nutrients useful for plants, generating advances and an increase in yield per hectare. Tannins provide phenolic compounds (96%) that improve root development, have elicitor properties and hinder nematodes (https://fertilgest.imagelinenetwork.com/dettaglio.formulato.cfm?lang=it&codice=35326& accessed on 23 January 2023).

Bortan was applied to rocket in fertirrigation once after 10 days of sowing at a dosage of 10 L/ha and at a concentration of 0.5 mL/L, as recommended by the manufacturer.

Rocket was harvested on June 16 (40 DAS) between 10 a.m. and 12 p.m. to reduce the effects of diurnal fluctuations in secondary metabolite content, according to Huseby et al. (2013) [48]. Successively, rocket leaves were closed in Ziploc bags and transported to the laboratory. Leaves were washed thoroughly with water and stored at −80 °C for further analyses. The experimental design consisted of one complete randomized block per treatment, arranged with three replicates per treatment.

### 3.2. Physical Parameters

The color of the leaves was evaluated using a Minolta colorimeter (CR5, Minolta Camera Co., Osaka, Japan), used to determine L* (lightness), a* (green to red) and b* (blue to yellow) chromaticity.

The dry weight was assessed on a representative sample of 30 leaves which were put in a laboratory oven at 60 °C until they were completely dry.

### 3.3. Photosynthetic Pigments

Chlorophyll a, b and total carotenoids were evaluated as described by Wellburn (1994) [49], using NN-dimethylformamide as solvent. Absorbance of the extract was measured at 480, 647 and 664 nm using a UV–VIS V-630 (Jasco, Milan, Italy). Results were expressed using the equations reported by Wellburn (1994) in relation to the fresh weight (FW) [49].

### 3.4. Bioactive Compounds

Rocket leaves (2:10; *w*/*v*) were mixed with a hydroalcoholic solution (methanol/water 80:20 *v*/*v*) and left to macerate for 12 h at room temperature in a dark room. After the incubation period, the samples were centrifuged at 14,000× *g* for 10 min, after which the hydroalcoholic supernatant was separated from the plant material. The total phenolic content (TP) was evaluated through the Folin–Ciocâlteu method described by Magri and Petriccione (2022), with some modifications. The hydroalcoholic extract (200 µL) was mixed with the Folin–Ciocalteu reagent and sodium carbonate (7.5% *w*/*v*). The reaction mix was incubated for 2 h in a dark room, after which absorbance at 765 nm was recorded. Results were calculated by using a calibration curve obtained though standard concentrations of gallic acid and expressed as mg of gallic acid equivalent (GAE) per 100 g^−1^ FW [50].

The total flavonoid content (TF) was determined in line with Magri et al. (2020) [51]. Results were expressed as mg of catechin equivalent (CE) per 100 g^−1^ FW. The reaction mixture contained 400 μL of the hydroalcoholic extract, sodium nitrite 5%, aluminum chloride 10% and sodium hydroxide 1 M. The results were collected at 510 after 15 min of dark incubation.

The antioxidant activity (AOX) was measured by 1,1-diphenyl-2-picryl-hydrazil (DPPH) according to Magri et al. (2020) and indicated as µmol Trolox equivalent (TE) g^−1^ of FW [51]. The mixture assay contained 75 μL of the extract and 63.4 μmol L^−1^ DPPH, and the decrease in absorbance was evaluated at 515 nm using a UV–VIS spectrophotometer (Model V-630, Jasco, Tokyo, Japan) after 10 min of dark incubation.

### 3.5. Ascorbic Acid Content

The ascorbic acid (AA) content was determined according to Goffi et al. (2020) with some modifications. Rocket leaves (2:5, *w*/*v*) were mixed with a solution of 16% (*v*/*v*) metaphosphoric acid and 0.18% (*w*/*v*) disodium ethylene diamine tetraacetic acid (Na-EDTA) [52]. To 200 μL of extract, 0.3% metaphosphoric acid (*v*/*v*) and Folin’s reagent (diluted at 1:5, *v*/*v*) were added. Results were expressed as mg of ascorbic acid (AA) per g^−1^ FW.

### 3.6. Sample Preparation and SPME Procedure

Volatiles profiling was achieved following the HS-SPME/GC–MS method reported by Cozzolino et al. (2016), using the DVB/CAR/PDMS (50/30 µm) fiber, and with an extraction temperature and extraction time of 40 °C and 20 min, respectively. For the sample preparation, 0.75 g of rocket leaves were put into a 20 mL HS vial with a screw cap (Supelco, Bellefonte, PA, USA) containing 0.5 mg of Na_2_SO_4_. In each sample, 5 mL of 5% ethanol (*v*/*v*) and 10 μL of 3-octanol (0.4 µg/mL), used as internal standard (IS), were included.

Each vial, sealed with a Teflon (PTFE) septum and an aluminum cap (Chromacol, Hertfordshire, UK), was placed in the instrument dry block-heater at 40 °C for 20 min for system equilibration. The extraction and injection steps were automatically executed using an autosampler MPS 2 (Gerstel, Mülheim, Germany). Successively, the fiber was directly introduced into the vial’s septum for 20 min, to allow for VOC adsorption onto the fiber surface.

### 3.7. Gas Chromatography–Quadrupole Mass Spectrometry Analysis (GC–qMS)

The SPME fiber was inserted into the injector port of the gas chromatographer GC 7890A (Agilent Technologies, Santa Clara, CA, USA) hyphenated with a mass spectrometer 5975 C (Agilent). Analytes were thermally desorbed and directly moved to a capillary HP-INNOwax column (30 m × 0.25 mm × 0.5 µm, Agilent) for separation.

The oven temperature was first set at 40 °C for 5 min, increased to 240 °C at 4 °C min^−1^ and kept at 240 °C for 5 min. The temperature of the ion source and of the quadrupole were set at 230 °C and 150 °C, respectively; He was used as carrier gas at a flow rate of 1.5 mL min^−1^; injector temperature was set at 240 °C and the pulsed splitless mode was set for the analysis.

The fiber was held in the injector for 10 min. Mass spectra were acquired at 70 eV and volatiles were recorded by a mass-selective detector. The detector worked in the mass range from 30 to 300 m/z with a scan rate of 2.7 scans/s. Analyses of individual samples were obtained in triplicate in a randomized sequence where blanks were also acquired.

Volatile compounds identification was determined by comparison of the experimental mass spectra with those available in standard NIST05/Wiley07 database libraries and by matching the retention times with those of an in-house-developed retention time library built on authentic compounds. Moreover, identification of VOCs was also performed by comparing their retention indices (RI) (as Kovats indices), calculated based on the retention time of a series of C_8_–C_22_ n-alkanes with linear interpolation, with those of commercial standards or from literature data. Each sample was analyzed in triplicate with a randomized sequence in which blanks were also recorded.

The areas of the detected volatiles were estimated from the total ion current (TIC) and the semi-quantitative data of each component (relative peak area, RPA%) were measured in relation to the peak area of 3-octanol (IS).

### 3.8. Statistical Analysis

Statistical significance between untreated samples and rocket leaves treated with plant-based biostimulant was detected according to a two-sample *t*-test using MetaboAnalyst v5.0. For each measurement, the mean values were significantly different (ns = not significant; ** significant for *p* ≤ 0.01; * significant for *p* ≤ 0.05).

A principal component analysis (PCA) was applied to describe the influence of treatments on physical, qualitative and aromatic traits in rocket leaves to identify the principal components contributing to the variation within the dataset. Statistical analysis was carried out using the SPSS software package, version 20.0 (SPSS Inc., Chicago, IL, USA).

## 4. Conclusions

The results obtained revealed that field application of Bortan, at the concentration used in this study, improved the quality traits of rocket samples, increasing the photosynthetic pigment content and the non-enzymatic antioxidant system. In addition, the biostimulant treatment significantly changed the pattern of VOC emissions in rocket leaves, suggesting the activation of different metabolic pathway which could cause diverse sensory characteristics between the treated and control samples. Nevertheless, the definition of the VOCs related to the sensory impression of the two investigated rocket samples needs further experiments, aiming at integrating the information of the chemical characterization of VOCs with the odor analysis by gas chromatography–olfactometry (GC–O), which is a valuable technology to clarify aroma-active volatiles and/or to investigate the relationship between aroma and taste in food samples.

Although the positive effects of biostimulants on the cultivation of vegetables and fruits have been extensively described, PBs are hardly employed into conventional cultivation practices. This could be due to the fact that farmers have insufficient information on the functions and usage of biostimulants, which results in a fear in facing higher cultivation costs and a decrease in crop productivity. Other concerns include selection among the multitude of preparations and the need to choose a suitable biostimulant for a particular plant variety to obtain the optimum results in term of plant quality and yield. In this context, results presented here could help to improve the knowledge of the effects of plant-based biostimulants on leafy vegetables. Nevertheless, further research should be carried out to confirm these preliminary data by enlarging sample size, by testing different concentrations of Bortan, by checking alternative application practices (i.e., watering vs. spraying) and by using other plant species/cultivars.

## Figures and Tables

**Table 1:** Color parameters and weight loss in untreated samples (Control) and in rocket leaves treated with Bortan (Treated).

Samples	L*	A*	B*	Chr	H°	WL (%)
Treated	39.97 ± 1.96	−8.23 ± 0.57	19.38 ± 1.40	21.00 ± 1.55	115.62 ± 0.90	97.80 ± 0.04
Control	34.59 ± 2.48	−7.19 ± 0.61	14.98 ± 1.29	16.62 ± 1.40	113.10 ± 0.46	97.80 ± 0.84
*p*	**	**	***	***	**	ns

For each measurement, the mean values are significantly different (ns = not significant; *** significant for *p* ≤ 0.001; ** significant for *p* ≤ 0.01;) according to Two-sample *t*-test using MetaboAnalyst v5.0.

**Table 2:** Bioactive compounds in the rocket leaves treated with Bortan (Treated) and in the untreated samples (Control).

Samples	TP (mg GAE 100 g FW)	TF (mg CE 100 g FW)	AA (mg AA 100 g FW)	AOX (µmol TE g FW)
Treated	91.94 ± 5.04	102.51 ± 7.00	16.63 ± 0.69	9.99 ± 0.20
Control	68.56 ± 3.45	81.56 ± 2.29	14.04 ± 0.05	8.61 ± 0.49
*p*	***	***	**	**

TP: total polyphenols; TF: total flavonoids; AA: ascorbic acid; AOX: antioxidant activity. For each measurement, the mean values are significantly different (*** significant for *p* ≤ 0.001; ** significant for *p* ≤ 0.01;) according to Two-sample *t*-test using MetaboAnalyst 5.0.

**Table 3:** Volatile compounds detected by HS-SPME/GC–MS in the rocket leaves treated with Bortan (Treated) and in the untreated samples (Control) and their identification codes.

Metabolites	Code	RIcal/RIt	ID	Control	Treated	*p*
**Ketones**						
3-Pentanone	K1	980/980	RI/MS/S	2.86	4.07	**
1-Penten-3-one	K2	1026/1026	RI/MS/S	4.66	15.55	**
3-Octanone	K3	1272/1272	RI/MS/S	7.46	15.41	**
6-Methyl-5-hepten-2-one	K4	1350/1348	RI/MS/S	3.30	0.00	**
**Aldehydes**						
Hexanal	Ald1	1084/1086	RI/MS/S	11.45	14.38	**
*cis*-3-Hexanal	Ald2	1148/1148	RI/MS	6.45	58.64	**
2-Hexenal	Ald3	1242/1248	RI/MS/S	245.48	722.66	**
Octanal	Ald4	1309/1308	RI/MS/S	1.79	0.00	**
Nonanal	Ald5	1401/1401	RI/MS/S	6.83	1.18	**
2,4-Heptadienal	Ald6	1401/1401	RI/MS/S	0.62	2.59	**
Decanal	Ald7	1506/1505	RI/MS/S	7.86	10.08	**
**Alcohols**						
1-Penten-3-ol	Al1	1188/1189	RI/MS/S	18.53	48.78	**
*cis*-2-penten-1-ol	Al2	1272/1272	RI/MS/S	0.00	21.60	**
1-Hexanol	Al3	1340/1339	RI/MS/S	8.56	6.55	**
*trans*-3-Hexen-1-ol	Al4	1367/1366	RI/MS/S	8.65	2.76	**
*cis*-3-Hexen-1-ol	Al5	1374/1374	RI/MS/S	382.28	148.21	**
*trans*-2-Hexen-1-ol	Al6	1394/1394	RI/MS/S	60.91	23.61	**
2,6-Dimethylcyclohexanol	Al7	-	MS/S	1.14	0.00	**
Terpinen-4-ol	Al8	1609/1609	RI/MS/S	1.53	0.00	**
**Glucosinolate Hydrolysis Products (GHPs)**						**
Methyl ITC	GHP1	1282/1278	RI/MS/S	6.30	5.55	*
3-Butenyl ITC	GHP2	1479/1463	RI/MS	2.41	0.00	**
Pentyl ITC	GHP3	1511/1540	RI/MS	15.32	3.13	**
4-Methylpentyl ITC	GHP4	1487/1490	RI/MS	84.32	70.32	**
Hexyl ITC	GHP5	1582/1588	RI/MS/S	18.59	15.35	**
3-Methylthiopropyl ITC	GHP6	1970/1979	RI/MS/S	11.55	10.28	**
Benzyl ITC	GHP7	2109/2109	RI/MS/S	7.03	1.90	**
2-Ethylphenyl ITC	GHP8	2230/2233	RI/MS	4.53	2.21	**
**Others**						**
2-Ethylfuran	O1	960/965	RI/MS/S	4.72	7.60	**
1-Pentene-3-ethyl-2-methyl	O2	-	MS/S	5.46	12.29	**
*cis*-3-Hexen-1-ol acetate	O3	1320/1320	RI/MS/S	38.09	1.64	**
Anisole	O4	1350/1350	RI/MS/S	10.34	10.43	ns
5-Methylhexanenitrile	O5	1362/1358	RI/MS	2.54	5.61	**

RIsp: Relative retention indices calculated against n-alkanes (C8–C20) on HP-INNOwax column; RIt: Relative retention indices on polar column reported in the literature by www.pherobase.com, www.flavornet.org and www.ChemSpider.com, accessed on 23 January 2023. Identification method indicated by the following: RI: Kovats retention index on HP-INNOwax column; MS: NIST and Wiley libraries spectra; S: co-injection with authentic standard compounds on the HP-INNOwax column. Semi-quantitative data of each component (Relative Peak Area, RPA%) were measured in relation to the peak area of 3-octanol (IS). For each measurement, the mean values are significantly different (ns = not significant; ** significant for *p* ≤ 0.01; * significant for *p* ≤ 0.05) according to Two-sample *t*-test using MetaboAnalyst 5.0.
